# Integrative chloroplast omics in citrus: computational insights into genomic structural adaptation and phylogenetic relationships from 35 species

**DOI:** 10.3389/fpls.2026.1769665

**Published:** 2026-01-29

**Authors:** Su Lin, Jinfang Shu, Chengnan Kang, Wenxin Fang, Xingxing Liang, Kai Xu, Haijie Ma

**Affiliations:** 1 Key Laboratory of Quality and Safety Control for Subtropical Fruit and Vegetable, Ministry of Agriculture and Rural Affairs, Collaborative Innovation Center for Efficient and Green Production of Agriculture in Mountainous Areas of Zhejiang Province, College of Horticulture Science, Zhejiang Agricultural and Forestry (A&F) University, Hangzhou, Zhejiang, China; 2 School of Grassland Science, Beijing Forestry University, Beijing, China

**Keywords:** chloroplast genome, citrus, codon usage bias, comparative genomics, phylogeny, simple sequence repeats, structural variation

## Abstract

The chloroplast genome serves as a valuable tool for plant phylogenetic studies due to its conserved structure and slow evolutionary rate. Despite the economic importance of the genus *Citrus*, comprehensive comparative analyses of chloroplast genomes across multiple species remain limited. As one of the ancestors of *Citrus*, *Citrus medica L*. serves as a key representative in studies of the origin and evolution of the genus. In this study, we conducted a comprehensive comparative analysis of chloroplast genomes from 35 Citrus species and their close relatives, with *C. medica* as a focal species, to investigate structural variation, codon usage patterns, and phylogenetic relationships. All genomes exhibited a typical quadripartite structure, ranging from 159 to 161 kb with GC contents of 38.41-38.49%. While genome synteny was highly conserved, expansions and contractions at IR boundaries provided species-specific variation. SSR analysis revealed abundant mononucleotide repeats with a strong AT bias, predominantly distributed in non-coding regions. Codon usage analysis indicated a preference for A/U-ending codons, and ENC-GC3s, neutrality plot, and PR2 analyses suggested that natural selection was the main force shaping codon usage bias. Most protein-coding genes were under strong purifying selection, whereas *matK* and *rps16* exhibited elevated Ka/Ks ratios, suggesting relaxed selective constraints or potential signals of positive selection. Phylogenomic analysis strongly supported the monophyly of *Citrus* and resolved intrageneric relationships, grouping species into distinct ancestral and cultivated clades. Overall, this study provides essential chloroplast genomic resources for molecular breeding, species identification, and understanding *Citrus* adaptation.

## 1 Introduction

Chloroplasts, the photosynthetic organelles of plant cells, possess their own genome that has proven to be an invaluable tool for plant evolutionary studies (Dobrogojski et al., 2020). The chloroplast genome typically exists as a circular DNA molecule characterized by a highly conserved quadripartite structure- comprising large and small single-copy regions separated by inverted repeats (Bausher et al., 2006b; Dobrogojski et al., 2020; He et al., 2024). This genome encodes approximately 110–130 genes primarily involved in photosynthesis, transcription, translation, and various metabolic processes (Jansen et al., 2007; Yan et al., 2025). Beyond its functional role, the chloroplast genome exhibits several features that make it particularly valuable for evolutionary research, including predominantly uniparental inheritance and a relatively slow evolutionary rate compared with nuclear DNA. In addition, gene content and gene order are generally conserved across related species (Daniell et al., 2016, 2021). These features, combined with the development of efficient sequencing technologies, have established chloroplast genomics as an essential tool for investigating plant phylogenetics and molecular evolution across diverse taxonomic levels (Zhou et al., 2021).

Beyond their role in photosynthesis, chloroplast genomes serve as powerful molecular systems for addressing a wide range of evolutionary questions. Several intrinsic properties contribute to their utility in evolutionary biology. The high copy number per cell facilitates DNA extraction and sequencing (Takamatsu et al., 2018); while the conserved genome structure enables reliable comparative analyses across distant taxa. In addition, uniparental inheritance provides valuable insights into lineage-specific evolutionary histories. These characteristics have made chloroplast genomes particularly valuable for reconstructing robust phylogenetic frameworks, especially in taxonomically challenging groups where nuclear genomes show complex patterns of hybridization and introgression (Fang et al., 2018). Furthermore, the varying evolutionary rates across different regions of the chloroplast genome, from highly conserved rRNA genes to more rapidly evolving intergenic spacers, allow researchers to address phylogenetic questions at multiple taxonomic levels, from deep divergences to recent speciation events (Clegg et al., 1994). The presence of structural variations, such as expansions/contractions of inverted repeats and gene rearrangements, provides additional phylogenetic markers and insights into genome evolution processes. Beyond phylogenetic applications, chloroplast genomes offer valuable data for population genetics, species identification, and understanding molecular evolutionary processes through analyses of codon usage bias and selective pressures (Zhao et al., 2018; Chakraborty et al., 2020). These versatile applications, coupled with advancing sequencing technologies, have positioned chloroplast genomics as an essential component of modern plant evolutionary studies.

The genus Citrus presents a particularly compelling case for the application of chloroplast genomics, given its complex evolutionary history and substantial economic importance (de Araújo et al., 2003; Curk et al., 2015). As one of the world’s most widely cultivated fruit crops, citrus species have undergone extensive natural and artificial hybridization, resulting in intricate phylogenetic relationships that have challenged taxonomic classification for decades (Wang et al., 2023). Previous molecular studies, while providing valuable insights, have often relied on limited chloroplast markers or restricted taxonomic sampling, leaving significant gaps in our understanding of chloroplast genome evolution across the genus. The availability of complete chloroplast genome sequences for an increasing number of citrus species now enables comprehensive comparative analyses that can address fundamental questions regarding genome structure conservation, sequence variation patterns, and evolutionary dynamics (Shi et al., 2023). Furthermore, the maternal inheritance of chloroplast genomes in citrus offers a distinct perspective for tracing maternal lineages and understanding the role of specific wild species in the domestication and diversification of cultivated varieties (Wang et al., 2022). This is particularly relevant for clarifying the contributions of fundamental species such as citron (*Citrus medica*), mandarin (*Citrus reticulata*), and pummelo (*Citrus maxima*) to the formation of modern citrus cultivars through complex hybridization events (Wu et al., 2018b).

Recent investigations into citrus chloroplast genomes have revealed several fundamental characteristics while simultaneously highlighting significant knowledge gaps. Studies have established that citrus chloroplast genomes typically range between 159–161 kb in length and maintain the conserved quadripartite structure common to angiosperms (Shivakumar et al., 2017). Research on specific species, including sweet orange (*Citrus sinensis*) and pummelo (*C. maxima*), has identified structural variations at IR boundaries, particularly involving the *ycf1* and *trnH* genes, suggesting their potential utility as phylogenetic markers (Carbonell-Caballero et al., 2015). However, these investigations have primarily focused on economically significant cultivars, with wild relatives and ancestral species remaining substantially underrepresented. Furthermore, existing studies have largely emphasized structural features while paying insufficient attention to evolutionary patterns such as codon usage bias and selective constraints (Zhang et al., 2021; Geng et al., 2022; Hu et al., 2024), despite their importance in understanding molecular adaptation. The limited species sampling in previous comparative analyses has constrained our ability to reconstruct comprehensive phylogenetic relationships and understand genome evolution across the entire genus. These limitations underscore the necessity for an expanded analysis encompassing a broader taxonomic representation to fully elucidate the evolutionary dynamics of chloroplast genomes in *Citrus* and resolve persistent taxonomic uncertainties through robust phylogenetic inference.

To address these limitations, this study presents a comprehensive analysis of 35 complete chloroplast genomes representing the phylogenetic diversity of *Citrus*. We employ an integrated approach to examine genome structure variations, SSR distribution patterns, codon usage evolutionary mechanisms, and selective pressures on protein-coding genes. Through advanced phylogenomic analyses, we reconstruct robust evolutionary relationships within the genus. This research provides systematic insights into chloroplast genome evolution in *Citrus*, establishing a reliable phylogenetic framework to inform taxonomic revisions and guide future breeding strategies and germplasm utilization.

## 2 Materials and methods

### 2.1 Plant material collection and DNA sequencing

Fresh leaves of *C. medica*. were collected from the Citrus Research Institute in Chongqing, China. Plant materials were cultivated under controlled greenhouse conditions at 25 ± 2°C with 60-70% relative humidity and a 16/8-hour light/dark photoperiod. High-quality genomic DNA was extracted from the leaf tissues using a modified cetyltrimethylammonium bromide (CTAB) protocol. Sequencing libraries were prepared following the manufacturer’s guidelines and subjected to paired-end (150 bp) sequencing on the DNBSEQ-T7 platform (MGI Tech) (Liu et al., 2025), generating approximately 20 GB of raw data. This achieved approximately 100× coverage depth for the chloroplast genome. For comparative analysis, publicly available sequences of 34 additional Citrus species were retrieved from the NCBI database (https://www.ncbi.nlm.nih.gov/).

### 2.2 Chloroplast genome assembly and annotation

The complete chloroplast genome of *C. medica*. was assembled using a seed-based *de novo* strategy implemented in NOVOPlasty (v4.3), a software specifically designed for organelle genomes. Initial annotation was conducted with the GeSeq online platform under default parameters, with reference to published *Citrus* chloroplast genomes to improve accuracy. The genome structure was further manually curated using NCBI’s Sequin tool to verify start/stop codons and exon-intron boundaries. Finally, a circular genome map was generated using the online tool Chloroplot (https://irscope.shinyapps.io/Chloroplot/), illustrating the characteristic quadripartite structure and spatial distribution of genes within the chloroplast genome.

### 2.3 SSR analysis

SSR analysis was performed on the 35 citrus chloroplast genomes using IMEx v2.1 with minimum repeat thresholds set at 10 for mononucleotide, 5 for dinucleotide, 4 for trinucleotide, and 3 for tetra-, penta-, and hexanucleotide repeats (Mudunuri and Nagarajaram, 2007). Detected SSRs were systematically classified by repeat unit length (mono-, di-, tri-, tetra- to hexanucleotide), genomic location (coding sequences, introns, or intergenic spacers), and nucleotide composition (AT-rich or GC-rich). The distribution patterns of conserved SSRs were visualized using the heatmap package in R to assess their potential association with IR boundary variations.

### 2.4 RSCU analysis

Relative Synonymous Codon Usage (RSCU) analysis was employed to systematically evaluate codon usage bias in the chloroplast genomes. Following the extraction of all protein-coding sequences, RSCU values for each synonymous codon were computed using Python scripts according to the standard formula: RSCU = observed frequency of a codon/expected frequency under equal usage across all synonymous codons for that amino acid. As established in previous studies, an RSCU value of 1.0 indicates no usage preference (Barkley et al., 2006), values >1.0 represent preferentially used codons, and values <1.0 indicate low-frequency usage (Liu et al., 2020). The resulting RSCU patterns were visualized through a heatmap generated with the heatmap package in R, utilizing a discrete color gradient (ranging from ≤0.5 to 2.5–3.0). This method accounts for variations in amino acid composition, thereby enabling accurate characterization of codon preference patterns at the genome level (Perrière and Thioulouse, 2002).

### 2.5 ENC analysis

The Effective Number of Codons (ENC), a measure of synonymous codon usage bias independent of amino acid composition, was calculated to assess the overall magnitude of codon usage bias in chloroplast protein-coding genes. The ENC values were computed using the formula proposed by Wright: ENC = 2 + 9/F_2_ + 1/F_3_ + 5/F_4_ + 3/F_6_, where F_2_ through F_6_ represent the bias factors for amino acids with 2 to 6 synonymous codons, respectively. This metric ranges from 20 (extreme bias) to 61 (no bias), with lower values indicating stronger codon usage bias (Wright, 1990). Simultaneously, the GC content at the third synonymous codon position (GC3s) was calculated for each gene. The relationship between ENC and GC3s was visualized in a scatter plot, where the theoretical expected curve (assuming codon usage is determined solely by GC3s composition) was included for reference. The distribution of data points relative to this expected curve was analyzed to assess the relative contributions of mutational pressure and translational selection in shaping codon usage patterns.

### 2.6 Neutrality plot analysis

Neutrality plot analysis was conducted to assess the relative contributions of mutational pressure and natural selection in shaping codon usage patterns. For each protein-coding gene, the GC contents at the first and second codon positions (GC12) and at the third synonymous position (GC3) were calculated. The relationship between GC12 and GC3 was examined through linear regression analysis, with regression slopes and coefficients of determination (R²) serving as key indicators. A regression slope approaching 1 suggests that mutational pressure predominantly influences codon usage, while a slope approaching 0 indicates that natural selection is the dominant evolutionary force. This analytical approach effectively distinguishes between neutral evolution and selective constraints by comparing the nucleotide composition constraints at different codon positions (Tang et al., 2021; Yang et al., 2023).

### 2.7 PR2 plot analysis

Parity Rule 2 (PR2) plot analysis was performed to evaluate the relative contributions of mutation bias and natural selection to synonymous codon usage by examining the balance between complementary nucleotides at the third codon position. The base composition at the third codon position was analyzed by calculating A_3_/(A_3_+T_3_) and G_3_/(G_4_+C_3_) ratios for each protein-coding gene. These values were plotted in a scatter diagram with G_3_/(G_3_+C_3_) as the x-axis and A_3_/(A_3_+T_3_) as the y-axis (Yang et al., 2023). The central point (0.5, 0.5) represents the theoretical equilibrium where A=T and G=C in the absence of selection pressure. Deviation from this central point indicates base composition bias, with points distributed in the four quadrants revealing specific nucleotide usage patterns. Data points were colored according to citrus varieties and faceted by species to enhance comparative analysis. This method effectively distinguishes the relative contributions of mutational bias and natural selection to codon usage patterns through quantitative assessment of base composition bias.

### 2.8 Correspondence analysis

Correspondence analysis (COA) was employed to investigate the major trends in synonymous codon usage variation across the chloroplast protein-coding genes of Citrus species. The analysis was based on the relative synonymous codon usage (RSCU) matrix, where each coding sequence was represented as a 59-dimensional vector (excluding methionine AUG, tryptophan UGG, and the three stop codons). Dimensionality reduction was performed using the FactoMineR package in R, extracting the first two principal axes that account for the largest proportion of variation in codon usage. The distribution of genes along these axes was visualized using the factoextra package, revealing clustering patterns and major trends in codon usage. This multivariate analysis not only identifies the primary sources of variation in codon usage among genes but also helps detect potential influencing factors, such as functional constraints or evolutionary pressures, that shape codon preference in citrus chloroplast genomes (Perrière and Thioulouse, 2002).

### 2.9 Collinearity analysis

A systematic comparative analysis was conducted on the chloroplast genomes of 35 species to elucidate structural conservation and evolutionary relationships. Genomic visualization was performed using the genoPlotR package in R (v4.1.0), with all annotation data processed through customized R scripts. Gene annotations were extracted from GFF files and converted into standardized tables, followed by color-coding according to functional categories (e.g., photosynthesis, ATP synthesis, transcription, and translation). Sequence similarity was assessed through pairwise BLASTN alignments, and the results were converted into visualization objects. A maximum likelihood phylogenetic tree in Newick format was integrated to provide an evolutionary context. The analysis revealed the typical quadripartite structure of *Citrus* chloroplast genomes, with gray connecting lines illustrating conserved syntenic regions where color intensity corresponds to sequence similarity. This approach provided evidence for understanding structural variations, including rearrangements and inversions, during the evolutionary history of *Citrus* species.

### 2.10 Selection pressure analysis

Selection pressure on chloroplast protein-coding genes in *Citrus* was evaluated using the ratio of nonsynonymous (Ka) to synonymous (Ks) substitution rates (Ka/Ks) (Wu et al.), a widely used metric for inferring evolutionary constraints on coding sequences. Ka, Ks, and Ka/Ks values for homologous gene pairs were calculated using KaKs_Calculator v2.0 (Wang et al., 2010). In general, Ka/Ks ratios greater than 1 may indicate positive selection, ratios less than 1 suggest purifying selection, and values close to 1 are consistent with neutral evolution. This approach provides a framework for assessing selective pressures acting on chloroplast protein-coding genes.

### 2.11 Phylogenetic reconstruction

Phylogenetic relationships among the *Citrus* species were reconstructed based on the concatenated sequences of 13 protein-coding genes (PCGs) from the chloroplast genomes. Multiple sequence alignment was performed using MAFFT v7.450 under the auto strategy (Katoh and Standley, 2013), and the resulting alignments were concatenated and trimmed using trimAl v1.4 with the “automated1” option. The best-fit partitioning scheme and nucleotide substitution models were selected using PartitionFinder V2.1.1 under the Bayesian Information Criterion (BIC) (Lanfear et al., 2016). Maximum likelihood (Jansen et al.) analysis was conducted with RAxML-NG v1.2.2 (Kozlov et al., 2019), employing 1000 bootstrap replicates to assess branch support. Bayesian inference (BI) was performed using MrBayes v3.2.7 with four independent Markov chain Monte Carlo (MCMC) runs, each for 1,000,000 generations and sampling every 1000 generations. The first 25% of samples were discarded as burn-in, and the remaining trees were used to generate a consensus phylogeny. This comprehensive approach enabled robust reconstruction of evolutionary relationships within the genus *Citrus*.

## 3 Results

### 3.1 Morphological and chloroplast genomic features of citrus

**Figure 1:**
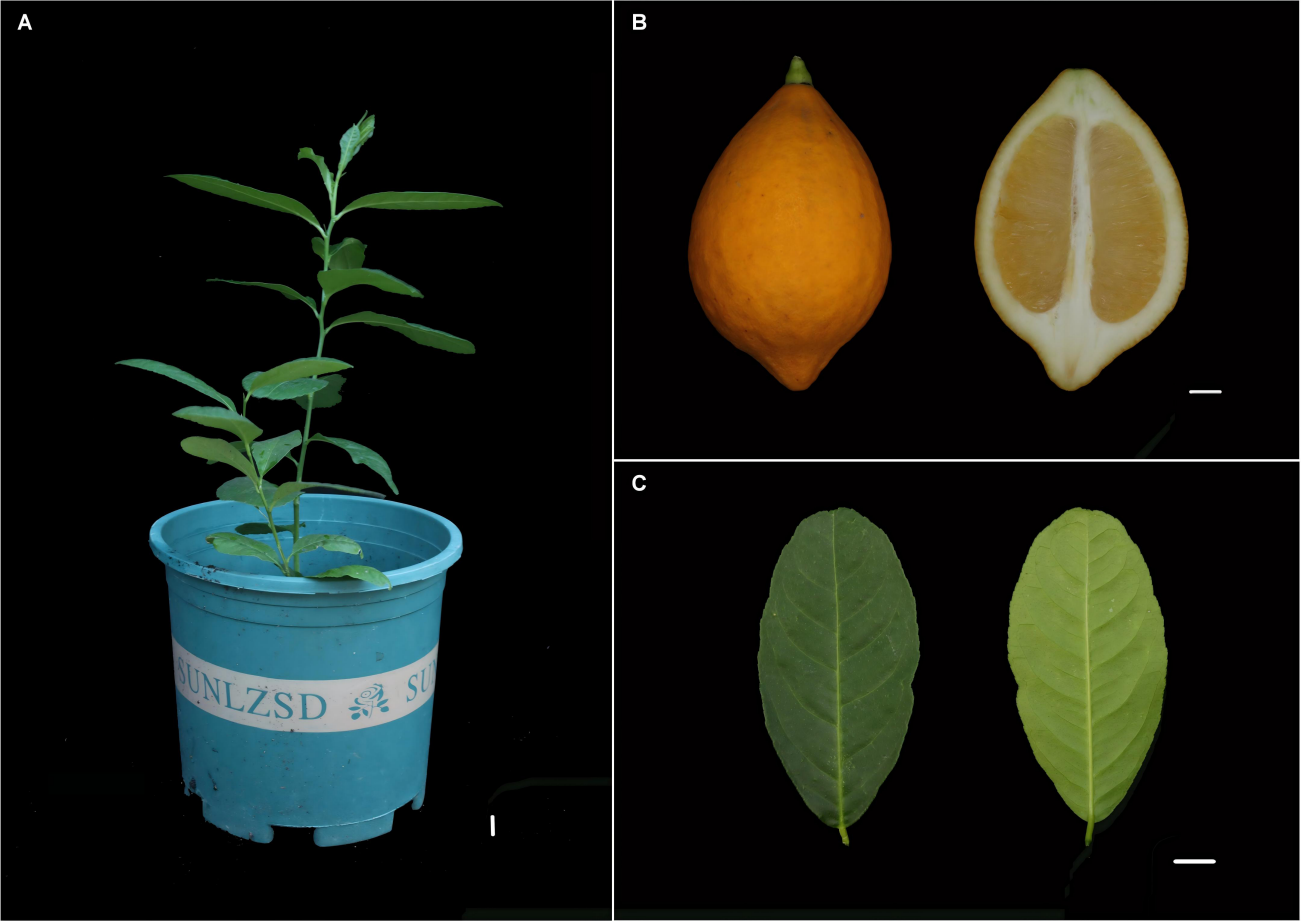
Morphological characteristics of *C*. *medica.***(A)** Overall growth habit of *C*. *medica*. **(B)** Different forms of fruits: a mature citron fruit (left) and a cross-section of the fruit (right). **(C)** Leaf specimen illustrating the characteristic ovate shape and prominent venation. Scale bar = 1 cm.

**Figure 2:**
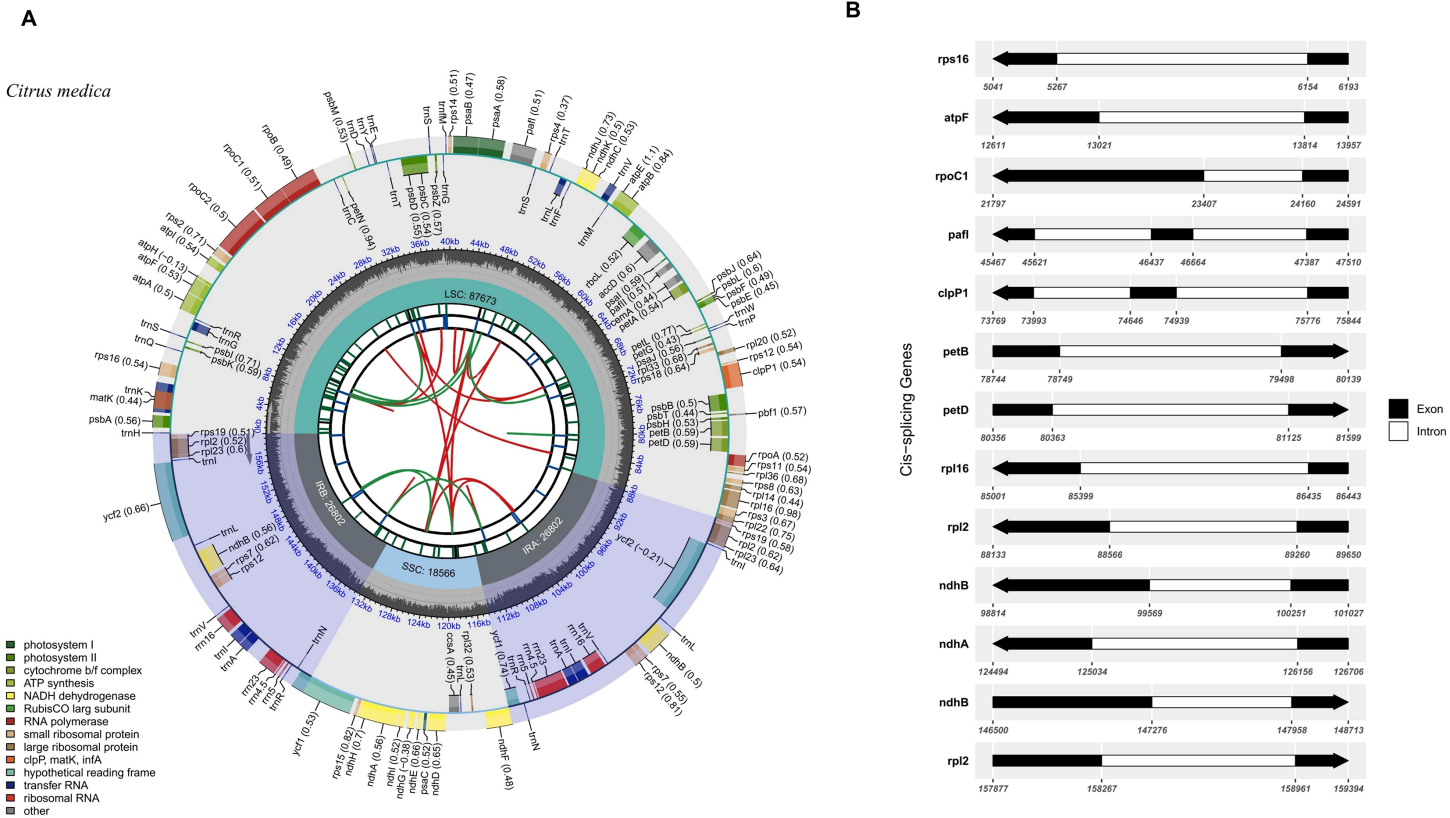
Complete chloroplast genome map of *C*. *medica.***(A)** Circular genome map of the *C*. *medica* chloroplast genome. Schematic representation of the complete chloroplast genome showing overall structural features. The map displays six concentric tracks from center outward: (1) dispersed repeats with direct (D) and palindromic (P) repeats connected by red and green arcs respectively; (2) long tandem repeats as blue bars; (3) short tandem repeats/microsatellites with color coding (black: complex repeats, green: mononucleotide, yellow: dinucleotide, purple: trinucleotide, blue: tetranucleotide, orange: pentanucleotide, red: hexanucleotide); (4) genome regions showing small single-copy (SSC), inverted repeats (*IRa and IRb*), and large single-copy (LSC) regions; (5) GC content variation along the genome; and (6) gene locations with functional classification indicated by colors. Genes transcribed clockwise and counterclockwise are shown on the outer and inner sides respectively. The genome is 159,843 bp in length with 38.45% GC content and contains 87 genes including 8 rRNAs and 37 tRNAs. **(B)** Cis-splicing gene structure map of the *C*. *medica* chloroplast genome. Schematic representation of genes containing introns arranged according to their genomic positions. Gene names are listed on the left, with corresponding exon-intron structures shown on the right. Black boxes represent exons and white boxes represent introns. Arrows indicate the transcriptional direction of each gene. Note that exon and intron lengths are not drawn to scale.

Citron (C. medica) is a small evergreen tree with distinct morphological features. The trunk is erect, covered with grayish-brown bark that exhibits rough texture and irregular longitudinal fissures. Current-year branches are green and relatively flexible, primarily responsible for photosynthesis and nutrient transport, while perennial branches become more lignified, turning yellowish-brown, and mainly function in structural support and nutrient storage (**Figure 1A**). The fruit of citron is a typical hesperidium. Mature fruits are oblong or elongated, with a thick and rough peel densely covered with oil glands. The color transitions from dark green in immature stages to light yellow or golden yellow at maturity. A longitudinal section of the fruit reveals a well-developed, thick white spongy layer (albedo) beneath the flavedo. Inside, multiple segments are arranged regularly, filled with pale yellow, elongated juice vesicles. Seeds are primarily embedded within these segments, showing typical localization (**Figure 1B**). The leaves are simple, alternate, and leathery in texture, generally exhibiting an oblong or ovate-lanceolate shape. The leaf base is cuneate, and the margin is entire or occasionally with inconspicuous serrations. The adaxial surface is dark green and glossy, while the abaxial surface is lighter in color with prominently visible oil glands. The midvein is distinctly raised on the abaxial side, and the secondary veins form a reticulate pattern, which is clearly observable. This leaf structure is closely associated with the synthesis and accumulation of aromatic compounds in the species (**Figure 1C**). The distinctive morphological traits of C. medica, including its thick fruit albedo, abundant oil glands, and leathery leaves, are closely associated with its specialized metabolic and physiological functions. These phenotypic characteristics provide a relevant biological context for subsequent chloroplast genome analyses because chloroplasts contribute to a certain extent to photosynthesis primary metabolism and the biosynthesis of precursor compounds for secondary metabolites. Therefore, integrating morphological observations with chloroplast genomic features facilitates a more comprehensive understanding of the evolutionary and functional characteristics of C. medica within the genus Citrus. The chloroplast genomes of Citrus species exhibited the typical quadripartite structure characteristic of angiosperms. Using C. medica as a representative example, the complete chloroplast genome was 159,843 bp in length with a GC content of 38.45% (**Figure 2A**). It consisted of a large single-copy (LSC) region of 87,673 bp, a small single-copy (SSC) region of 15,866 bp, and a pair of inverted repeat (IRa/IRb) regions, each 26,802 bp. Annotation identified a total of 131 genes, comprising 86 protein-coding genes, 8 rRNA genes, and 37 tRNA genes. The genomic map clearly illustrated the enrichment of photosynthesis-related genes (e.g., psbA, psbD) within the LSC region (**Figure 2A**; **Table 1**; **Supplementary Table S1**). The map also delineated the precise locations and distributions of genes across the four structural regions, while revealing the complex exon-intron structures of key chloroplast genes. Notably, several genes (e.g., petD, rps16) possessed long intronic regions, potentially involved in transcriptional regulation (**Figures 2A, B**). Comparative analysis of the chloroplast genomes from 35 Citrus species (**Table 1**) revealed interspecific length variations ranging from 159 to 161 kb, with GC content varying between 38.41% and 38.49% (mean 38.448%). The overall mean nucleotide composition across all species was 30.47% A, 31.08% T, 19.58% C, and 18.87% G, indicating a pronounced AT bias. Codon position-specific analysis showed a pattern of GC content distribution as GC1 (46.21%) > GC2 (38.38%) > GC3 (32.02%), with a particularly strong AT preference (collectively 67.98%) at the third codon position (**Supplementary Table S1**).

**Table 1:** Summary of chloroplast genome features in *Citrus* species.

No.	Species	Acc. no.	Length	GC%	Protein	rRNA	tRNA	Total gene
1	*Citrus* x *aurantiifolia*	KJ865401	159893	38.44	93	8	37	138
2	*Citrus platymamma*	KR259987	160121	38.48	89	8	37	134
3	*Citrus depressa*	LC218435	160090	38.49	88	8	43	139
4	*Citrus latipes*	LC794892	160145	38.49	91	8	45	144
5	*Citrus polytrifolia*	MK250977	160211	38.44	93	8	37	138
6	*Citrus* x *aurantium (*bitter orange*)*	MT106672	160140	38.48	90	8	37	135
7	*Citrus cavaleriei (*Ichang papeda*)*	MT880606	160996	38.46	89	8	37	134
8	*Citrus* x *limon*	MT880608	160141	38.48	89	8	37	134
9	*Citrus trifoliata (*trifoliate orange*)*	MW207297	160381	38.42	89	8	37	134
10	*Citrus* x *clementina*	MW207298	160702	38.42	88	8	36	132
11	*Citrus erythrosa*	MW722946	160120	38.48	88	8	38	134
12	*Citrus unshiu (*satsuma mandarin*)*	MZ324823	160699	38.42	88	8	37	133
13	*Citrus australasica*	MZ929414	160335	38.42	88	8	37	133
14	*Citrus japonica (*marumi kumquat*)*	OM773616	160228	38.42	89	8	37	134
15	*Citrus hindsii*	OM773617	160259	38.42	89	8	37	134
16	*Citrus junos (*yuzu*)*	ON065547	160612	38.45	88	8	37	133
17	*Citrus keraji (*keraji*)*	ON065548	160121	38.48	88	8	37	133
18	*Citrus madurensis*	ON065549	160229	38.42	88	8	37	133
19	*Citrus mangshanensis*	ON065550	160262	38.44	88	8	37	133
20	*Citrus oto*	ON065551	160127	38.48	88	8	37	133
21	*Citrus tachibana*	ON065552	160600	38.42	87	8	37	132
22	*Citrus tarogayo*	ON065553	160121	38.48	88	8	37	133
23	*Citrus* x *paradisi*	ON065554	160185	38.47	88	8	37	133
24	*Citrus micrantha*	ON597621	159928	38.44	95	8	37	128
25	*Citrus sinensis (*sweet orange*)*	ON641345	160121	38.48	87	8	37	132
26	*Citrus australis (*Australian round lime*)*	ON872190	160413	38.44	87	8	37	132
27	*Citrus indica*	ON872191	160312	38.42	88	8	37	133
28	*Citrus jambhiri (*rough lemon*)*	ON872192	160210	38.42	88	8	37	133
29	*Citrus limonia (*Rangpur lime*)*	ON872193	160723	38.41	88	8	37	133
30	*Citrus nobilis*	ON872195	160700	38.42	88	8	37	133
31	*Citrus tangerina*	ON872196	160699	38.42	88	8	37	133
32	*Citrus hystrix (*Makrut lime*)*	PQ149287	159893	38.44	87	8	37	132
33	*Citrus reticulata (*mandarin orange*)*	PV928963	160121	38.48	85	8	37	130
34	*Citrus maxima (*pomelo*)*	PV928964	160185	38.47	85	8	37	130
35	*Citrus medica (*citron*)*	PX404588	159843	38.45	86	8	37	131

### 3.2 SSR analysis

**Figure 3:**
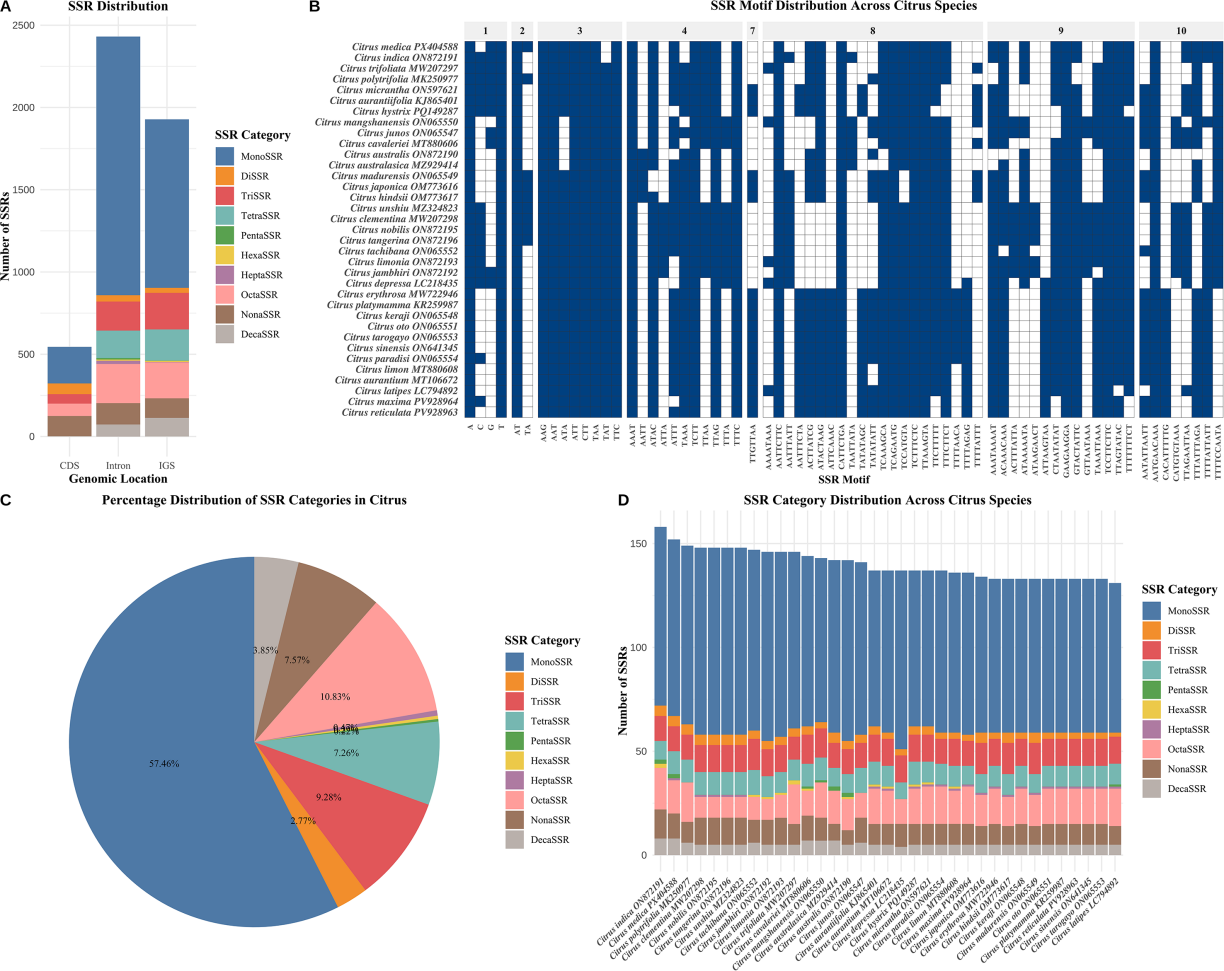
Comprehensive analysis of simple sequence repeats (SSRs) in chloroplast genomes of 35 *Citrus* species. **(A)** Distribution of SSR categories across different genomic locations including coding sequences (CDS), intergenic spacers (IGS), and other genomic regions. Bar heights represent the count of each SSR category with different colors indicating MonoSSR through ExtendedSSR types. **(B)** Heatmap displaying presence/absence patterns of SSR motifs across all analyzed *Citrus* species. Blue cells indicate presence (1) and white cells indicate absence (0) of specific SSR motifs. **(C)** Pie chart showing the percentage distribution of SSR categories identified across all species. Percentages are calculated based on total SSR count and represent the relative abundance of each category. **(D)** Stacked bar chart illustrating the distribution and abundance of SSR categories across individual *Citrus* species. Each bar represents one species with colors corresponding to different SSR categories. Species are ordered by total SSR count for better visualization of patterns.

Analysis of simple sequence repeats (SSRs) in the chloroplast genomes of 35 *Citrus* species revealed distinct distribution patterns. Mononucleotide repeats (MonoSSRs) were the most abundant type, accounting for 2,818 SSRs (57.46% of the total), followed by octanucleotide (OctaSSR, 531; 10.83%) and trinucleotide (TriSSR, 455; 9.28%) repeats. In contrast, pentanucleotide (PentaSSR, 11; 0.22%) and hexanucleotide (HexaSSR, 14; 0.29%) repeats were relatively rare, indicating a clear preference for specific repeat unit lengths in citrus chloroplast genomes (**Figures 3A, B**; **Supplementary Table S2**). A heatmap illustrating SSR motif distribution across species (**Figure 3C**) showed that A/T mononucleotide repeats were highly conserved and widespread among most species, reflecting their conserved nature within the genus. Species-specific motifs, such as ATAT and AATAT, were also identified, highlighting interspecific variation. Notably, SSR composition exhibited a significant AT-rich bias, consistent with the overall high AT content of citrus chloroplast genomes. The distribution of SSR types varied noticeably among citrus species (**Figure 3D**). For example, lemon (*C. limon*) had a significantly higher proportion of MonoSSRs compared to pummelo (*C. maxima*). Such diversity in SSR profiles reflects the dynamic evolution of SSR composition during species differentiation within the genus. Conservation analysis of SSR motifs (**Supplementary Table S2**) indicated that A/T mononucleotide repeats were present in nearly 100% of the examined species, suggesting possible functional or structural importance, whereas more complex repeat motifs exhibited species-specific distributions. Genomic localization analysis (**Figure 3A**; **Supplementary Table S2**) revealed a non-random distribution of SSRs: intronic regions contained the highest SSR density (49.57%), followed by intergenic spacers (IGS, 39.31%), while coding sequences (CDS) had the lowest density (11.11%). This pattern suggests that non-coding regions, under weaker selective constraints, are more prone to accumulate SSR variations. The scarcity of SSRs in coding regions likely reflects purifying selection against repeat sequences that could cause frameshift mutations or disrupt protein function. Importantly, the SSR loci identified in this study may serve as valuable molecular markers for species identification and phylogenetic studies in *Citrus*.

### 3.3 Analysis of relative synonymous codon usage

**Figure 4:**
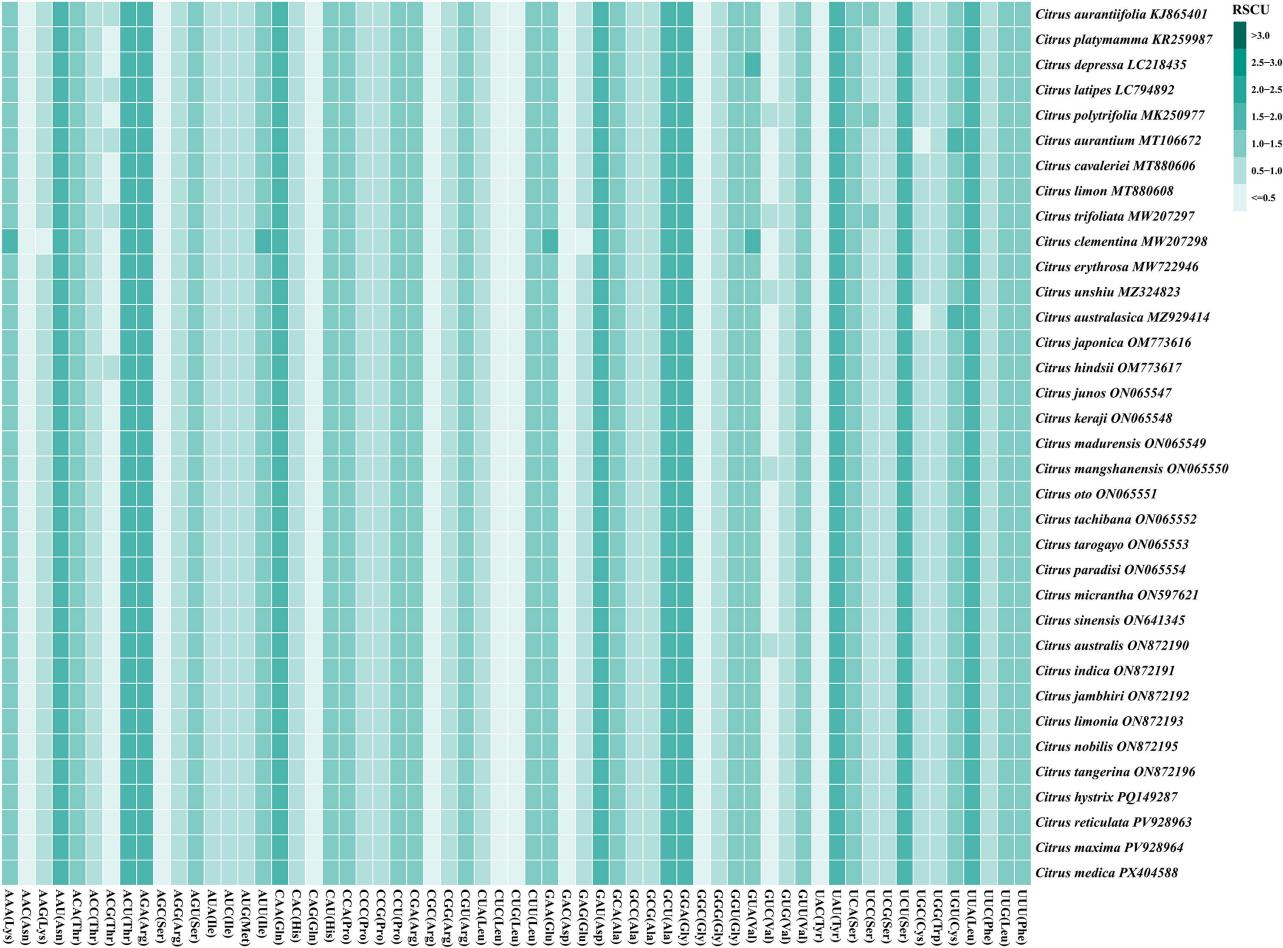
Heatmap visualization of Relative Synonymous Codon Usage (RSCU) patterns across 35 *Citrus* species chloroplast genomes. The heatmap displays RSCU values for all 61 sense codons (excluding stop codons) in chloroplast protein-coding genes. Each row represents a *Citrus* species, and each column represents a codon with its corresponding amino acid abbreviation in parentheses. Color intensity indicates RSCU values: darker blue colors represent higher values (preferred codons, RSCU > 1.0), while lighter colors represent lower values (under-represented codons, RSCU < 1.0). The color scale ranges from 0.33 to 1.96. This analysis reveals genus-wide patterns of codon usage bias in the Rutaceae family, providing insights into evolutionary constraints and translational efficiency in chloroplast genomes. Species are ordered phylogenetically to highlight evolutionary relationships and codon usage conservation patterns.

Analysis of Relative Synonymous Codon Usage (RSCU) was performed to investigate codon usage bias within the chloroplast genomes of the 35 *Citrus* species. Among the 20 standard amino acids encoded, tryptophan (*Trp*) and methionine were each specified by a single codon (UGG and AUG, respectively). The remaining 18 amino acids were encoded by 2 to 6 synonymous codons each (**Figure 4**; **Supplementary Table S3**). Comparative analysis of synonymous codon usage patterns across the 35 species revealed a remarkable degree of conservation within the genus. The RSCU heatmap demonstrated highly consistent usage patterns for the same codons across different *Citrus* species, indicating that codon preference in the chloroplast genome is largely conserved at the interspecific level. The analysis identified four codons with strong usage preference (RSCU > 1.6): UUA (Leu, average RSCU = 1.755), AGA (Arg, 1.734), GCU (Ala, 1.703), and UCU (Ser, 1.610). Conversely, 19 codons were significantly under-represented (RSCU < 0.6), including UAC (Tyr, 0.407), AGC (Ser, 0.419), and GGC (Gly, 0.435). An additional 13 codons displayed near-neutral usage (0.8 ≤ RSCU ≤ 1.2). These results systematically characterize the codon usage bias in the chloroplast genomes of *Citrus* species within the Rutaceae family, revealing a distinct pattern of preferential and avoided codon usage.

### 3.4 ENC-GC3s plot analysis

**Figure 5:**
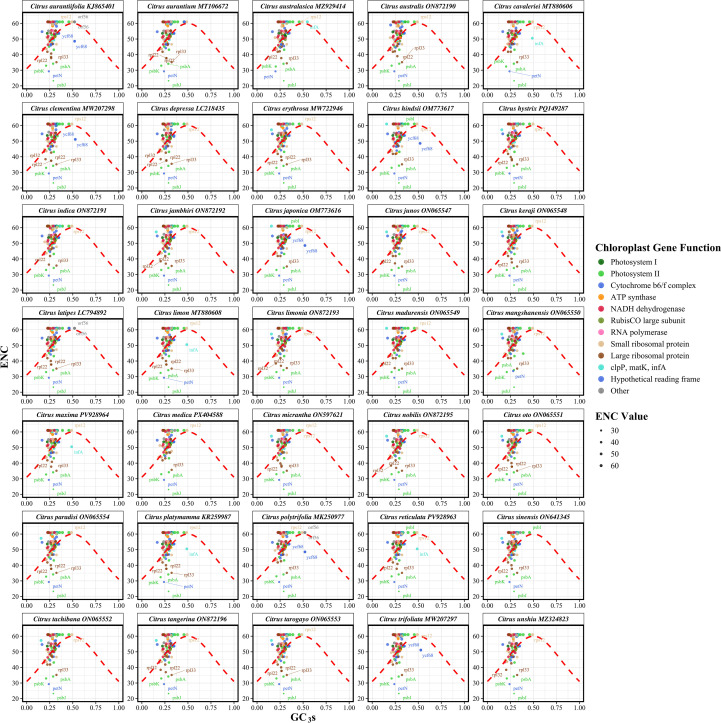
Analysis of synonymous codon usage bias in chloroplast genomes of 35 *Citrus* species. The effective number of codons is plotted against GC content at synonymous third codon positions (GC3s) for each species. Each point represents a protein-coding gene, with point size proportional to ENC values. The red dashed line shows the theoretical relationship between ENC and GC3s under neutral evolution [Wright’s curve: ENC = 2 + 29/(GC3s² + (1-GC3s) ²)], representing the expected values if codon usage is determined solely by base composition. Genes falling below this curve indicate additional selective forces beyond mutational bias affecting codon usage patterns. Different colors represent individual *Citrus* species to facilitate species identification and comparison of codon usage patterns.

To investigate the underlying evolutionary forces shaping codon usage bias in *Citrus* chloroplast genomes, an analysis of the relationship between the Effective Number of Codons and the GC content at the third synonymous codon position (GC3s) was conducted. Analysis of 35 representative *Citrus* species revealed ENC values ranging from 51.46 to 52.33 (mean = 51.93), while GC3s values varied between 0.2765 and 0.2921 (mean = 0.2823) (**Supplementary Table S4**). The ENC-GC3s scatter plot demonstrated that the vast majority of data points, each representing a protein-coding gene, were distributed above the theoretical expected curve (red dashed line in **Figure 5**), which represents the predicted relationship if codon usage were determined solely by GC3s composition. Genes from species such as *C. reticulata*, *C. ichangensis*, and *C. medica* exhibited distinct clustering patterns, suggesting that their chloroplast genes are subject to relatively uniform selective pressures. The observation that the measured ENC values were higher than the theoretical predictions based solely on GC3s provides evidence that natural selection, rather than mutational pressure, is the important force shaping codon usage bias in the chloroplast genomes of the *Citrus* genus. Notably, no significant differences in ENC-GC3s distribution patterns were observed between cultivated varieties (e.g., *C. sinensis*) and their wild relatives (e.g., *C. ichangensis*), implying that the process of artificial domestication has had a limited impact on codon usage preferences in the chloroplast genome.

### 3.5 Neutrality plot analysis

**Figure 6:**
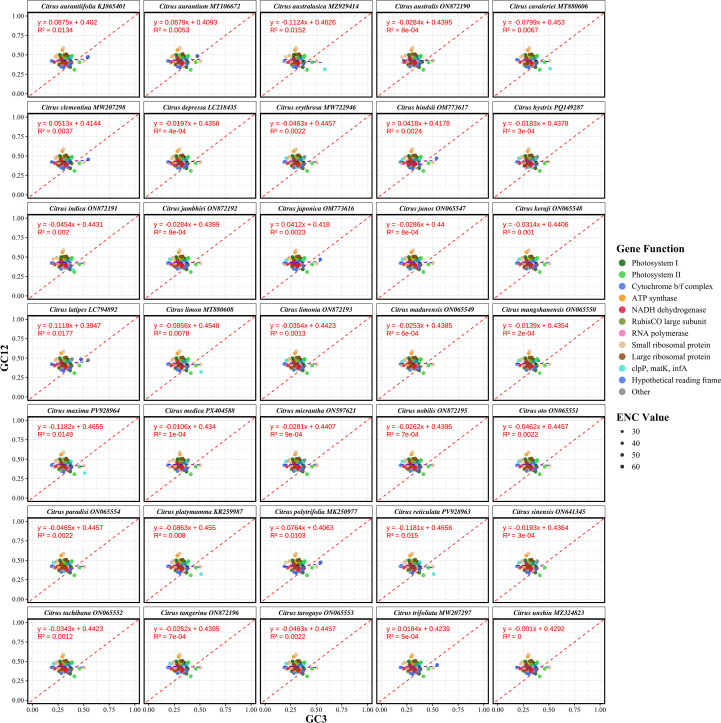
Neutrality plot analysis of synonymous codon usage patterns in 35 *Citrus* chloroplast genomes. Scatter plots depicting the relationship between GC content at the third codon position (GC3, x-axis) and the average GC content at the first and second positions (GC12, y-axis) for all protein-coding genes in each species. Blue dashed lines indicate species-specific linear regression lines; the red dashed line represents the neutral expectation (Y = X, slope = 1). Regression equations and R² values are provided in each panel. Species names are italicized according to taxonomic convention. The analysis demonstrates interspecific heterogeneity in codon usage evolution, with deviations from neutrality reflecting differential influences of mutational pressure and natural selection on synonymous codon usage.

Neutrality plot analysis was employed to further elucidate the relative contributions of mutational pressure and natural selection in shaping codon usage patterns in *Citrus* chloroplast genomes. This analysis examines the relationship between the GC content at the first and second codon positions (GC12) and the GC content at the third synonymous position (GC3). A regression slope approaching 1 suggests dominance of mutational pressure, whereas a slope approaching 0 indicates that natural selection is the primary evolutionary force. Analysis of the 35 *Citrus* species (**Figure 6**; **Supplementary Table S5**) revealed a mean GC12 content of 0.4303, which was significantly higher than the mean GC3 content of 0.3128. The data points were predominantly clustered within specific ranges for GC12 (0.4274–0.4321) and GC3 (0.3076–0.3226), indicating stringent selective constraints on nucleotide composition at different codon positions. The results of the regression analysis showed regression slopes fluctuating between -0.1182 and 0.1118, with a mean value of -0.0203. This minimal slope, coupled with low coefficients of determination (R²) ranging from 0 to 0.0177, mean = 0.0041), strongly supports the conclusion that natural selection, rather than mutational pressure, is the principal factor driving the formation of codon usage patterns in the chloroplast genomes of *Citrus* species within the Rutaceae family.

### 3.6 PR2 plot analysis

**Figure 7:**
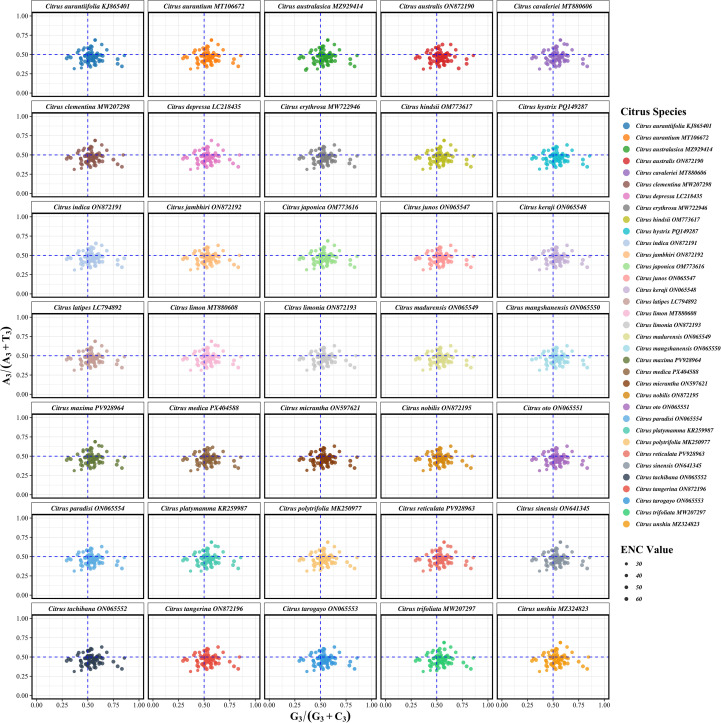
Parity Rule 2 (PR2) analysis of chloroplast protein-coding genes in 35 *Citrus* species. The comprehensive analysis shows the relationship between G_3_/(G_3_+C_3_) and A_3_/(A_3_+T_3_) ratios across 35 *Citrus* taxa representing the complete evolutionary diversity of the economically important citrus genus. Points are colored according to gene functional categories following CPGView standard classification: Photosystem I (dark green), Photosystem II (light green), Cytochrome b/f complex (royal blue), ATP synthase (dark orange), NADH dehydrogenase (crimson), RubisCO large subunit (olive drab), RNA polymerase (hot pink), Small ribosomal protein (burlywood), Large ribosomal protein (saddle brown), *clpP/matK/infA* (turquoise), Hypothetical reading frame (royal blue), Transfer RNA (black), Ribosomal RNA (medium purple), and Other genes (gray). Point size corresponds to ENC values. Dashed blue lines indicate the 0.5 threshold.

Parity Rule 2 (PR2) analysis was conducted to dissect the evolutionary forces influencing codon usage bias by examining the base composition at the third codon position. This analysis plots the relationship between A_3_/(A_3_+T_3_) and G_3_/(G_3_+C_3_) ratios for each protein-coding gene. The central point (0.5, 0.5) represents the theoretical equilibrium where A = T and G = C in the absence of any mutational or selection bias. The analysis revealed a highly consistent nucleotide usage pattern across all 35 *Citrus* species (**Figure 7**). The majority of data points were clustered around the central region of the plot but were slightly skewed towards the lower quadrants, indicating a minor but consistent preference for T over A and for G over C at the third codon position. Notably, the distribution patterns were remarkably similar across different *Citrus* species, sharing nearly identical central points and distribution ranges, which suggests that the mechanisms driving codon usage bias are evolutionarily conserved within the genus. Quantitative analysis confirmed this slight but stable deviation from equilibrium, with mean G_3_/(G_3_+C_3_) and A_3_/(A_3_+T_3_) ratios of 0.5373 and 0.4686, respectively (**Supplementary Table S6**). The observed imbalance in A/T and G/C usage demonstrates that codon usage patterns in *Citrus* chloroplast genomes are not solely governed by mutation but are significantly constrained by natural selection. This selective pressure might be linked to the optimization of photosynthetic efficiency in adapting to subtropical environments, potentially manifesting more strongly in photosynthesis-related genes. Consistent with findings from the ENC and neutrality plot analyses, no substantial differences were detected between cultivated varieties and wild species, reinforcing the notion that artificial domestication has exerted minimal influence on chloroplast codon usage preferences.

### 3.7 Correspondence analysis of synonymous codon usage

**Figure 8:**
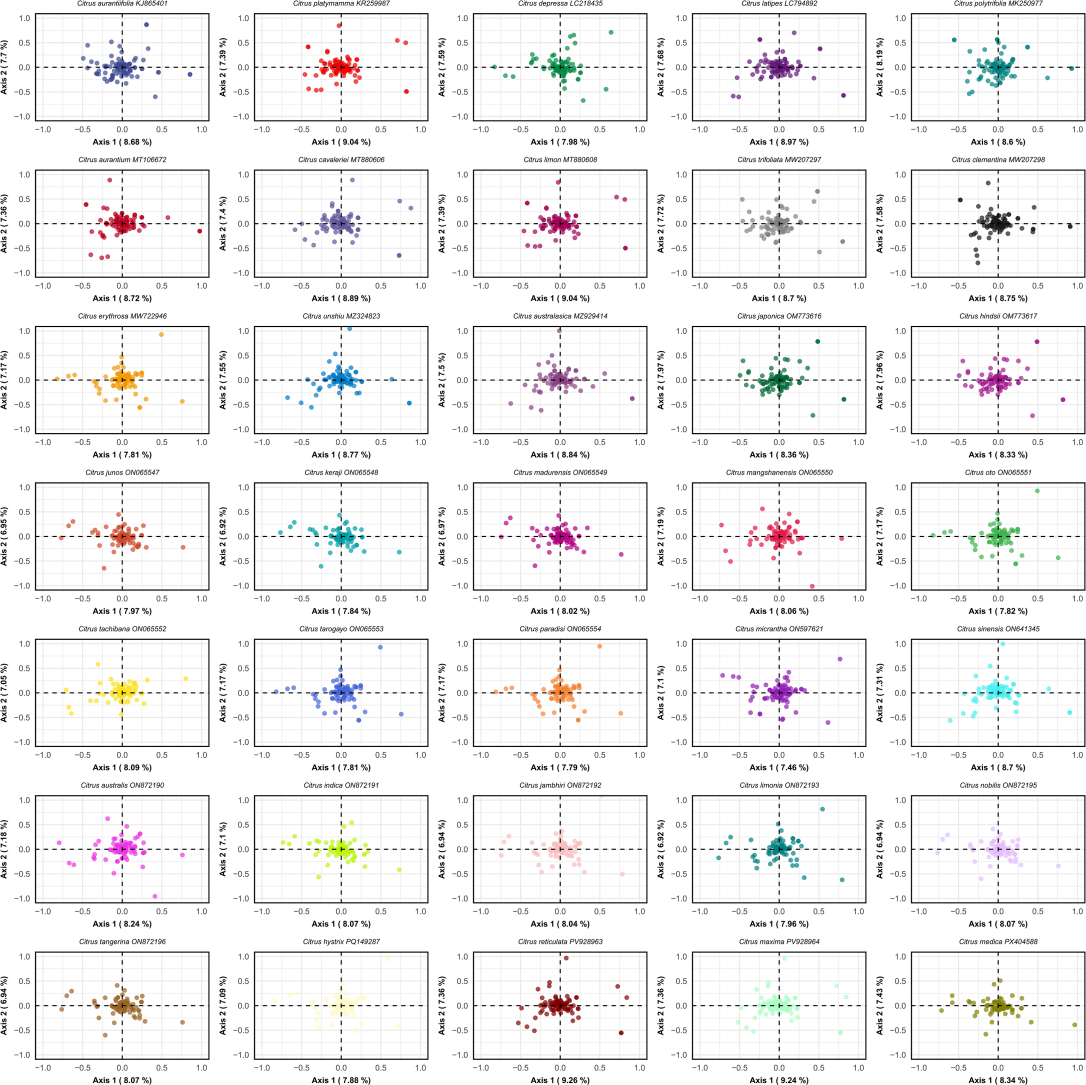
Correspondence Analysis (COA) of synonymous codon usage patterns in *Citrus* chloroplast genomes. The analysis presents two-dimensional COA plots for 35 *Citrus* species representing major commercial cultivars and wild species across the economically important *citrus* genus. Each panel shows the distribution of protein-coding genes in the space defined by the first two COA axes, which capture the primary patterns of codon usage variation. Points represent individual genes, with their positions reflecting similarities and differences in synonymous codon usage patterns. The percentage values on each axis indicate the proportion of total variance explained by that dimension.

Correspondence analysis (COA) was performed to visualize the multivariate variation in synonymous codon usage patterns across the chloroplast genomes of the studied *Citrus* species. The analysis was based on the relative synonymous codon usage (RSCU) matrix, with dimensionality reduction yielding the first two principal axes that capture the major trends in codon usage variation. The results revealed a highly consistent distribution pattern across all *Citrus* species, with gene data points forming a tight cluster near the origin of the COA plot (**Figure 8**). The first two principal axes collectively accounted for a relatively low proportion of the total variation (axis 1 = 8.349%, axis 2 = 7.326%, cumulative = 15.675%) (**Supplementary Table S7**). This low explanatory power suggests that codon usage patterns in *Citrus* chloroplast genomes are influenced by multiple factors collectively, rather than being dominated by a single determinant. The tight clustering of genes from all species indicates a high degree of homogeneity in codon usage among genes within the *Citrus* genus. Notably, A/U-ending and G/C-ending codons were distributed evenly across the four quadrants without clear separation based on third-position nucleotide composition. This pattern further corroborates that codon usage is primarily shaped by selective pressures rather than simple mutational biases. While minor distributional differences were observed in some species (e.g., *C. unshiu* and *C. aurantium*), the overall structural consistency suggests that chloroplast genomes in the genus have been subject to similar selective constraints throughout their evolutionary history. These highly conserved distribution patterns, indicative of strong selective constraints on codon usage preference, are consistent with the findings from the ENC plot, neutrality plot, and PR2 analyses. Together, they provide convergent evidence supporting the dominant role of natural selection in shaping codon usage patterns in *Citrus* chloroplast genomes. The absence of significant distributional differences between cultivated and wild species in the COA further implies that artificial selection during domestication has had minimal impact on shaping chloroplast codon usage preferences. These characteristics may be closely associated with the optimization of photosynthetic performance in adaptation to subtropical environments.

### 3.8 Patterns of selection pressure across chloroplast genes

**Figure 9:**
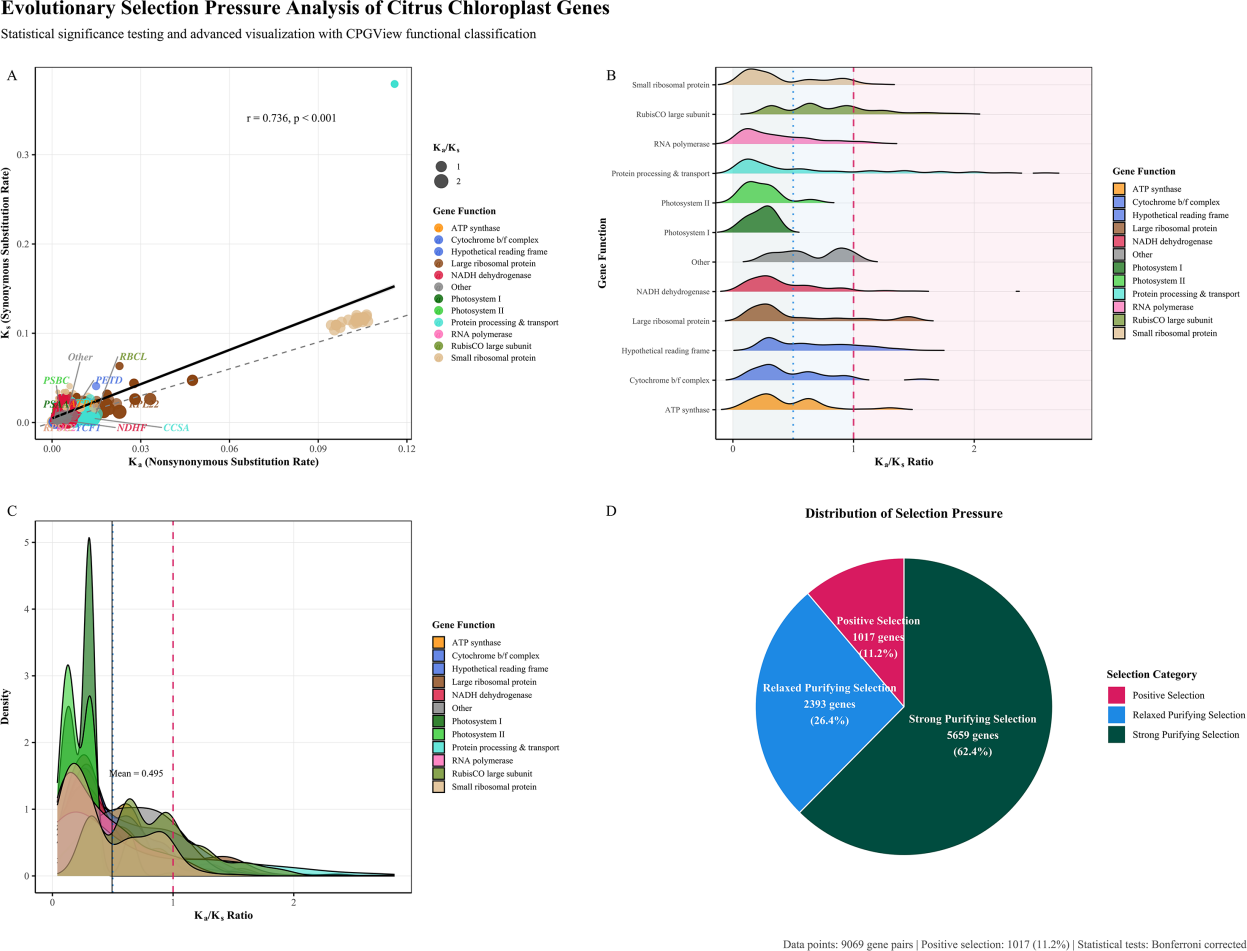
Evolutionary selection pressure analysis of *Citrus* chloroplast genes based on Ka/Ks ratios. **(A)** Scatter plot of Ka versus Ks rates colored by gene functional categories. Point size reflects Ka/Ks ratio magnitude. Diagonal dashed line indicates neutral evolution (Ka = Ks). Regression line with 95% confidence interval shows overall correlation trend. Gene names are labeled for representatives with highest Ka/Ks ratios in each functional category. **(B)** Ridge density plots showing Ka/Ks ratio distributions across gene functional categories. Background shading indicates selection pressure zones: strong purifying selection (green, Ka/Ks < 0.5), relaxed purifying selection (blue, 0.5 ≤ Ka/Ks ≤ 1.0), and positive selection (red, Ka/Ks > 1.0). **(C)** Density curves of Ka/Ks ratios by gene functional categories with overall mean indicated. Vertical lines mark selection pressure thresholds at Ka/Ks = 0.5 and 1.0. **(D)** Pie chart showing the distribution of selection pressure categories across all analyzed gene pairs. Gene functional categories are based on CPGView classification system. Total analyzed gene pairs: 9069. Genes under positive selection: 1017 (11.2%). Statistical significance testing performed with Bonferroni correction for multiple comparisons.

A comprehensive analysis of selection pressure, measured by the ratio of non-synonymous (Ka) to synonymous substitution rates (Ka/Ks), was performed on homologous chloroplast gene sequences across 35 *Citrus* species. The distribution analysis indicated that the vast majority of chloroplast genes are under purifying selection, consistent with their crucial roles in photosynthesis and cellular maintenance (**Figure 9**). Scatter plot analysis of Ka versus Ks values revealed a strong positive correlation (R² = 0.82, p < 0.001), with most genes clustering below the diagonal line representing neutral evolution (Ka = Ks). Notably, several genes, including *matK*, *rps16*, *ndhF*, and *ycf2*, exhibited relatively elevated Ka/Ks ratios compared with other chloroplast genes. Given the generally conserved nature of chloroplast genomes, these elevated ratios are more appropriately interpreted as indicative of relaxed selective constraints or potential signals of positive selection, rather than definitive evidence of adaptive evolution. In contrast, genes encoding core photosynthetic components consistently showed low Ka/Ks values, reflecting strong purifying selection and functional conservation. (**Figure 9A**). Density analysis of Ka/Ks ratios across different gene families (**Figure 9B**) revealed a distinct bimodal distribution pattern: a primary peak below 0.5 and a secondary peak extending beyond the neutral threshold. This bimodal characteristic suggests the coexistence of two different selective regimes in the *citrus* chloroplast genome—conservative evolution of core photosynthetic functions alongside adaptive evolution in specific genomic regions. Quantitative assessment of selection pressure categories across the entire dataset showed that 5,659 genes (62.4% of the dataset) were under strong purifying selection (Ka/Ks < 0.5), 2,393 genes (26.4%) experienced relaxed purifying selection (0.5 ≤ Ka/Ks ≤ 1.0), and 1,017 genes (11.2%) showed signals of positive selection (Ka/Ks > 1.0) (**Figure 9D**). This pattern is consistent with a predominance of purifying selection, where synonymous sites evolve faster than non-synonymous sites due to reduced functional constraints. These findings provide novel perspectives for understanding the adaptive evolution of chloroplast genomes in the genus *Citrus*.

### 3.9 Collinearity analysis

**Figure 10:**
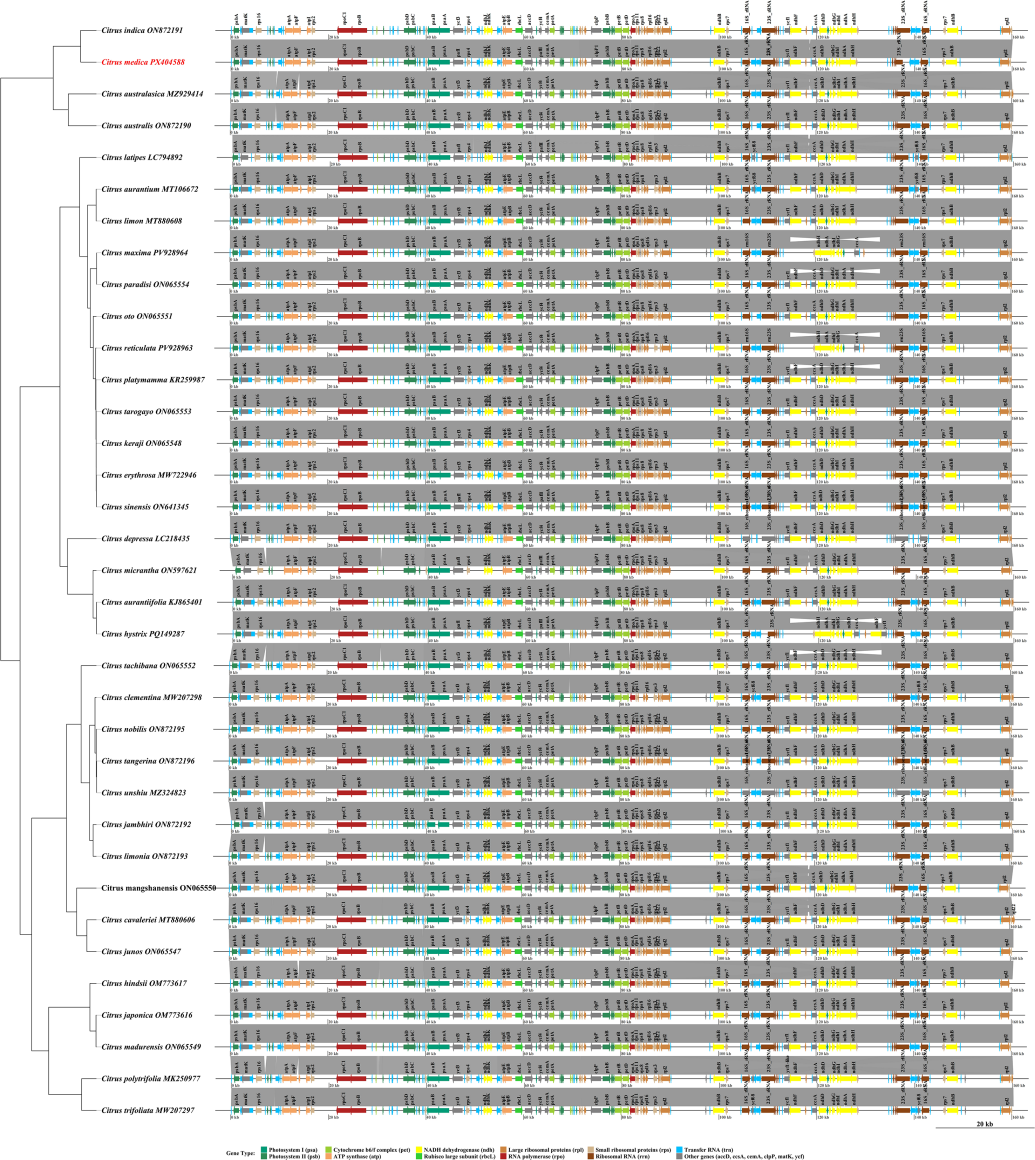
Comparative synteny analysis of chloroplast genomes across 35 *Citrus* species. Linear maps of chloroplast genomes are arranged alongside a phylogenetic tree (left), reflecting evolutionary relationships. Gene blocks are colored based on CPGView functional categories: Photosystem I (teal green), Photosystem II (sea green), Cytochrome b_6_/f complex (yellow green), ATP synthase (sandy brown), NADH dehydrogenase (yellow), RubisCO large subunit (lime green), RNA polymerase (fire brick), Small ribosomal proteins (tan), Large ribosomal proteins (peru), Transfer RNA (deep sky blue), Ribosomal RNA (saddle brown), and Other genes including *clpP, matK, and ycf* genes (gray). Conserved syntenic regions are connected by gray lines. The phylogenetic tree reflects established morphological and molecular evolutionary relationships within *Citrus*.

Collinearity analysis of the chloroplast genomes across the 35 *Citrus* species revealed a highly conserved genomic structural framework. Genes encoding proteins essential for photosynthesis, including those for photosystem I (psa), photosystem II (psb), the cytochrome *b*/*f* complex (pet), and ATP synthase (atp), were found to maintain an entirely conserved order and arrangement in all species, reflecting the strong selective pressure to preserve photosynthetic function (**Figure 10**). However, phylogenetically specific structural variations were observed in the inverted repeat (IR) boundary regions. The *ycf1* gene exhibited varying degrees of distribution across the IR/SSC junction among different species, and the positions of some tRNA genes, such as *trnH*-GUG, also showed interspecific divergence. These structural variations carried distinct phylogenetic signals: basal lineages, such as *C. mangshanensis* and *C. ichangensis*, retained ancestral structural features, whereas major cultivated varieties, including sweet orange (*C. sinensis*) and mandarin (*C. reticulata*), shared similar derived characteristics at the IR boundaries. Notably, the observed expansion/contraction events in the IR boundary regions showed a high degree of congruence with the established phylogenetic relationships within the genus. This consistency provides novel genomic evidence for understanding the evolutionary history of *Citrus* species and underscores the utility of IR boundary dynamics as informative markers for phylogenetic inference.

### 3.10 Phylogenetic analysis of citrus species

**Figure 11:**
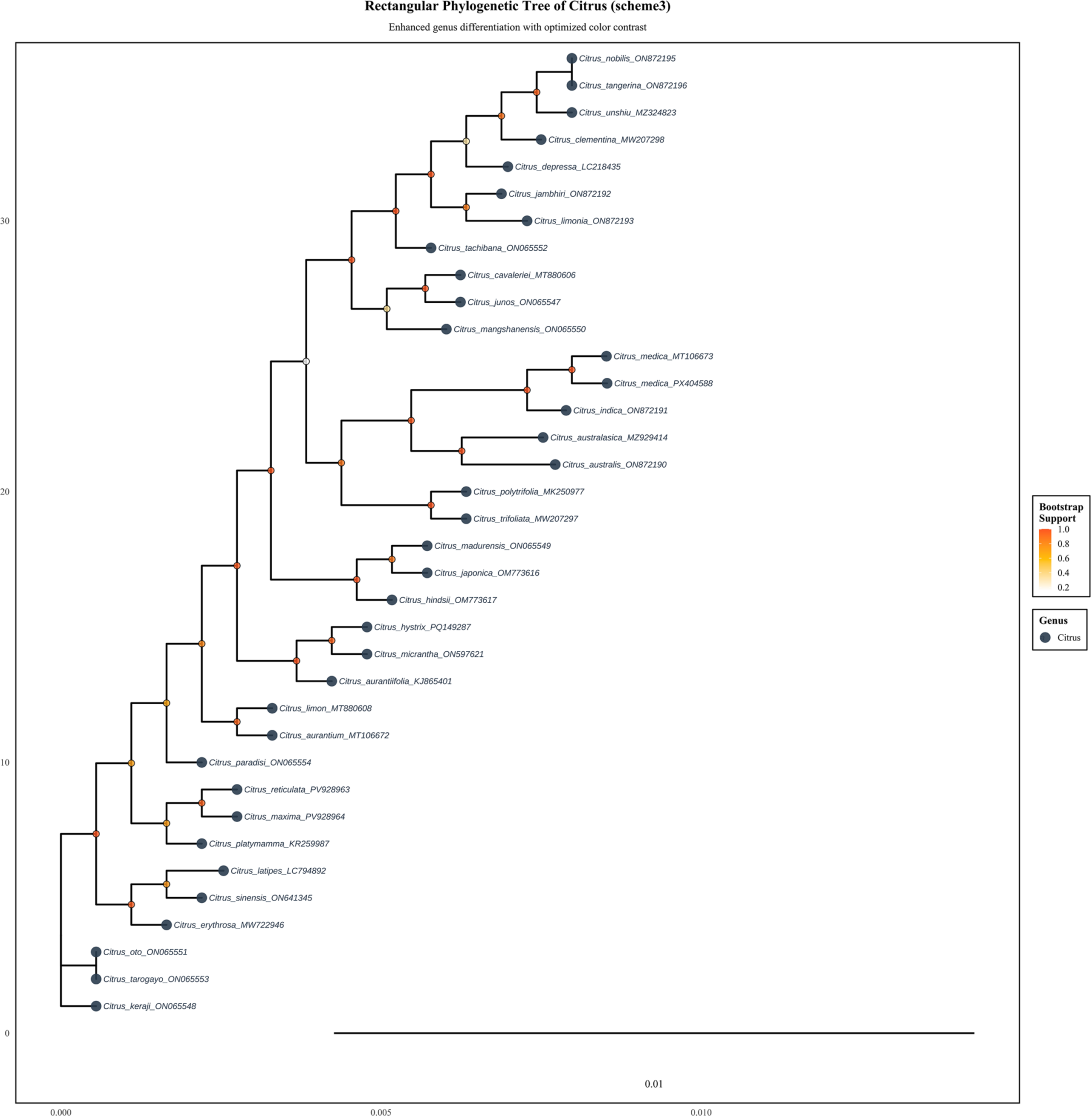
Phylogenetic tree of *Citrus* species based on chloroplast protein sequences. The tree represents evolutionary relationships among 36 *Citrus* species reconstructed using maximum likelihood analysis. Bootstrap support values are indicated by node coloration. Scale bar represents 0.1 substitutions per site.

**Figure 12:**
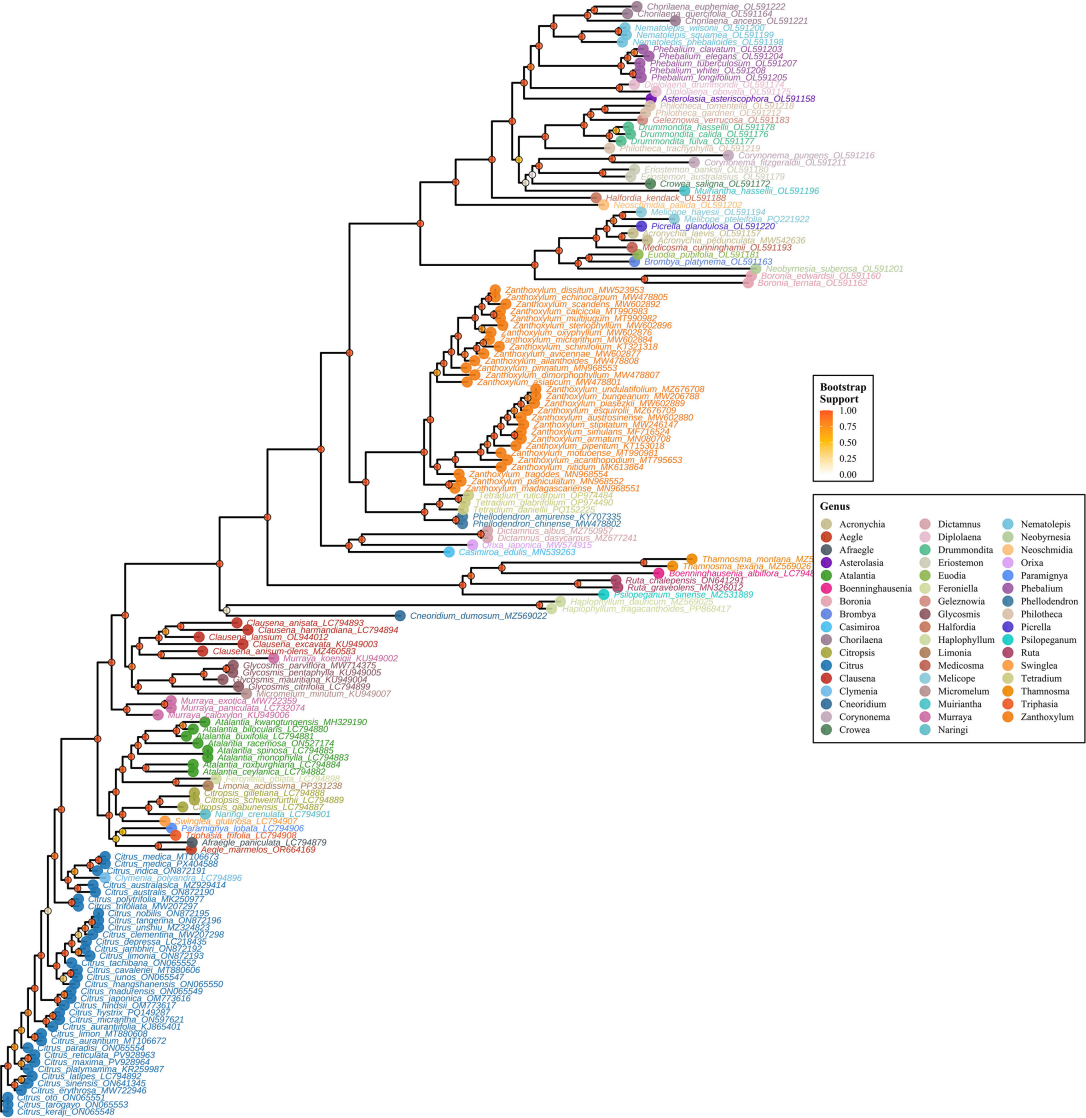
Phylogenetic tree of Rutaceae species with genus-level classification. The maximum likelihood phylogenetic reconstruction shows evolutionary relationships among Rutaceae species based on chloroplast protein sequence analysis. Colored circles at branch tips indicate genus membership, while node circles represent bootstrap support values with color intensity corresponding to statistical confidence. This rectangular layout provides clear visualization of branch lengths and evolutionary distances, facilitating detailed examination of phylogenetic relationships within and among genera in the citrus family. The tree topology reveals major evolutionary lineages and supports current taxonomic classifications within Rutaceae.

Phylogenomic reconstruction based on the concatenated sequences of chloroplast protein-coding genes elucidated the evolutionary relationships within *Citrus* and its close relatives in the Rutaceae family (**Figure 11**, **Figure 12**). The maximum likelihood tree strongly supported the monophyly of the genus *Citrus*, with all citrus species forming a distinct clade clearly separated from other genera within the family. Within the genus *Citrus*, the rectangular phylogenetic tree resolved several distinct evolutionary lineages with high statistical support, as indicated by the bootstrap values provided (ranging from 0.2 to 1.0). (1) A clade encompassing ancestral citrus species, including *C. mangshanensis*, received strong bootstrap support (>0.8); (2) The cultivated citrus clade was further subdivided, with pummelo (*C. maxima*) clustering independently, while mandarin (*C. reticulata*) and sweet orange (*C. sinensis*) formed a separate monophyletic group with robust statistical support; (3) A citron-related clade was identified, comprising lime (*C. aurantifolia*). Notably, the phylogenetic topology revealed clear evolutionary correspondences between cultivated varieties and their wild relatives, which aligns with the tree’s design purpose of enhanced genus differentiation via optimized color contrast. Hybrid-derived varieties, such as grapefruit (*C. × paradisi*), were positioned between their putative parental species in the tree. Furthermore, wild species from geographically proximate regions, such as *C. reticulata* from Yunnan and Guangxi, showed closer phylogenetic relationships. The phylogenetic analysis indicates that while *Citrus* constitutes a well-defined monophyletic group, species diversification occurred relatively recently in evolutionary time. The hierarchical clustering pattern observed in the phylogenetic tree is largely congruent with traditional morphological classifications, providing molecular evidence for taxonomic revision and germplasm utilization in *Citrus*.

## 4 Discussion

As the organelle responsible for photosynthetic energy conversion, the chloroplast retains structural and sequence features that record both conservative functional constraints and lineage-specific evolutionary changes (Arnon, 1966). By integrating structural, compositional, and evolutionary evidence from chloroplast genomes, this study provides new insights into the evolutionary mechanisms and speciation patterns within the genus *Citrus*.

The chloroplast genomes of *Citrus* exhibit the typical angiosperm quadripartite structure, consisting of a large single-copy (LSC) region, a small single-copy (SSC) region, and a pair of inverted repeat regions (IRa and IRb) (Wicke et al., 2011; Daniell et al., 2016). Their overall structure and gene order are highly consistent with other genera in Rutaceae (such as *Poncirus* and *Fortunella*) and various dicot families (e.g., Fabaceae, Solanaceae, and Brassicaceae), reflecting the high evolutionary conservation of chloroplast genomes (Bausher et al., 2006b; Shen et al., 2024; Chen et al., 2025). Nevertheless, structural variations exist at the IR boundaries, such as positional differences of the *trnH-GUG* gene at the LSC/IRb junction and the cross-boundary distribution of the *ycf1* gene at the SSC/IRa junction (Sato et al., 1999; Drescher et al., 2000). These dynamic changes at IR boundary regions are increasingly recognized as phylogenetically informative features and may serve as valuable structural markers for resolving evolutionary relationships within *Citrus*, as reported in previous comparative studies (Bausher et al., 2006a; Oueslati et al., 2016; Wang et al., 2021; Shi et al., 2023). A recent study on *Citrus* and related genera also highlighted that IR/SC junction variations are phylogenetically informative and can help delineate species boundaries.

Analysis of simple sequence repeats (SSRs) indicated that chloroplast SSR composition in *Citrus* is dominated by mononucleotide repeats, a pattern commonly observed in angiosperm plastid genomes (Barik et al., 2016). This bias toward simple, AT-rich repeat motifs reflects both underlying nucleotide composition and relaxed selective constraints in non-coding regions (El zayat et al., 2021).SSR loci were generally AT-rich across species, consistent with the overall high AT content of chloroplast genomes. Interspecific differences in SSR distribution, such as those between lemon (*C. limon*) and pummelo (*C. maxima*), reflect the dynamic evolution of SSR composition during species differentiation (García-Lor et al., 2012). Furthermore, SSRs were non-randomly distributed, with higher densities in intronic and intergenic spacer regions, suggesting that these non-coding regions, under weaker selective constraints, are more prone to accumulate variations (Peng et al., 2011). These polymorphic SSR loci not only serve as practical markers for species identification but also offer new avenues for phylogenetic reconstruction and genetic diversity analysis in *Citrus*, complementing earlier germplasm characterization efforts using nuclear SSR markers (Curk et al., 2015). From an applied perspective, the chloroplast SSRs identified in this study, particularly the highly conserved A/T-rich mononucleotide repeats, represent promising molecular markers for species identification, germplasm evaluation, and phylogeographic studies in Citrus. Compared with nuclear SSRs, chloroplast SSRs are characterized by maternal inheritance, lower recombination rates, and higher structural conservation, making them especially suitable for tracing evolutionary lineages and resolving interspecific relationships at the genus level. These features highlight the potential utility of chloroplast SSRs as complementary tools to nuclear markers in citrus genetic research.

Integrated multi-method analyses—including RSCU, ENC-GC3s, PR2, neutrality plot, and correspondence analysis—consistently indicated that natural selection is the dominant evolutionary force shaping codon usage preferences in *Citrus* chloroplast genomes (Plotkin and Kudla, 2011). The remarkably low explanatory power of the first two axes in the COA (<16%) suggests that multiple selective factors, rather than a single dominant mutational bias, collectively influence codon usage. This mechanism is potentially linked to optimizing translational efficiency of photosynthesis- and metabolism-related genes, a pattern observed in other plant groups like *Oryza* (Chakraborty et al., 2020) and *Capsicum* (He et al., 2024). Selection pressure (Ka/Ks) analysis further revealed differences in evolutionary constraints among genes of different functional categories: core photosynthetic genes (e.g., *psbA*, *psbD*) and ATP synthase genes were under strong purifying selection (Ka/Ks < 0.5), highlighting their indispensable roles in energy conversion (Jansen et al., 2007). Although a subset of chloroplast genes exhibited Ka/Ks ratios greater than unity, it should be emphasized that Ka/Ks > 1 alone is insufficient to conclusively demonstrate positive selection, particularly in the context of chloroplast genomes, which are generally subject to strong functional constraints. Elevated Ka/Ks values observed in genes such as *matK*, *rps16*, and *ycf2* may instead reflect episodes of relaxed purifying selection or lineage-specific evolutionary dynamics (Hao da et al., 2010). Further confirmation of positive selection would require more sophisticated approaches, such as branch-site model analyses or experimental functional validation. Nevertheless, the observed heterogeneity in selective pressure provides valuable insights into differential evolutionary constraints acting on chloroplast genes during *Citrus* diversification (Cejudo et al., 2019; Singhal et al., 2023).

Collinearity patterns further support the high degree of structural conservation in Citrus chloroplast genomes, with the gene order of photosynthesis-related clusters (e.g., *psa*, *psb*, *pet*, and *atp* genes) being entirely consistent, reflecting evolutionary stability of core functions under strong selective pressure (Wicke et al., 2011). This high degree of synteny is a common feature observed across angiosperm chloroplast genomes. At the same time, phylogenetically informative expansion/contraction events were detected at IR boundaries, particularly in regions adjacent to *ycf1* and *trnH*, providing reliable structural variation markers for clarifying interspecific relationships (Shimizu et al., 2016). Phylogenetic reconstruction based on 13 protein-coding genes strongly supported the monophyly of *Citrus* and divided the genus into several well-supported evolutionary clades (e.g., ancestral lineages, pummelo-related, mandarin-related, and citron-related groups) (Wu et al., 2014). These results are congruent with classical taxonomy and recent genomic studies, offering a solid molecular framework for resolving phylogenetic relationships and revealing the hybrid origins of cultivated varieties. Our phylogeny corroborates the findings of Wu et al. (2018) (Wu et al., 2018a), which identified citron, mandarin, and pummelo as the three fundamental biological species. The positioning of hybrid-derived varieties like grapefruit (*C. × paradisi*) between their putative parental species provides further chloroplast genomic evidence for their hybrid origin, consistent with nuclear genomic data (Wu et al., 2025). Furthermore, the close relationship between wild species from geographically proximate regions (e.g., *C. reticulata* from Yunnan and Guangxi) suggests possible regional evolutionary trajectories or gene flow.

## 5 Conclusions

This study provides a comprehensive genomic resource and evolutionary analysis of chloroplast genomes across a broad representation of *Citrus* species. Our findings underscore the conserved yet dynamically evolving nature of the chloroplast genome, highlight the predominant role of natural selection in shaping codon usage, and identify specific genes and structural features that have contributed to lineage diversification. The robust phylogenetic framework established here, consistent with but also extending previous studies by incorporating a wider taxonomic sampling and integrated genomic features, not only clarifies taxonomic relationships but also facilitates future studies on molecular breeding, species identification, and understanding adaptive evolution in this economically vital genus. The identified SSRs, codon usage patterns, and positively selected genes offer valuable candidates for developing molecular markers and for functional studies aiming to understand chloroplast function in citrus adaptation and productivity.

## Data Availability

All data generated or analyzed during this study are included in this published article and its **Supplementary Data** files. The raw chloroplast genome sequence of Citrus medica L. was deposited in GenBank under accession number PX404588.

## Supplementary material

Supplementary Table 1 Summary of Chloroplast Genome Composition and Nucleotide Characteristics in 35 *Citrus* Species.

Supplementary Table 2 Distribution and Classification of Simple Sequence Repeats (SSRs) in *Citrus* Chloroplast Genomes.

Supplementary Table 3 Relative Synonymous Codon Usage (RSCU) Values of Protein-Coding Genes Across 35 *Citrus* Chloroplast Genomes.

Supplementary Table 4 ENC and GC3s Values for Chloroplast Protein-Coding Genes in 35 *Citrus* Species.

Supplementary Table 5 Results of Neutrality Plot Analysis (GC12 *vs*. GC3) for Chloroplast Genes in 35 *Citrus* Species.

Supplementary Table 6 Parity Rule 2 (PR2) Analysis of Base Composition at Third Codon Positions in *Citrus* Chloroplast Genomes.

Supplementary Table 7 Coordinates of Genes in Correspondence Analysis (COA) Space Based on Codon Usage in 35 *Citrus* Species.
